# Tn-seq screens in *Candida glabrata* treated with echinocandins and ibrexafungerp reveal pathways of antifungal resistance and cross-resistance

**DOI:** 10.1128/msphere.00270-25

**Published:** 2025-07-07

**Authors:** Timothy J. Nickels, Kyle W. Cunningham

**Affiliations:** 1 Department of Biology, Johns Hopkins University 228291 , Baltimore, Maryland, USA; University of Guelph, Guelph, Ontario, Canada

**Keywords:** echinocandin, resistance, *Candida glabrata*

## Abstract

*Candida glabrata* (also known as *Nakaseomyces glabratus*) is the second most common cause of candidiasis, next to *Candida albicans,* and is rising in prevalence due to intrinsic and acquired mechanisms of antifungal resistance. This study utilizes transposon mutagenesis and deep sequencing (Tn-seq) to explore mechanisms of resistance to four different echinocandins (micafungin, anidulafungin, rezafungin, and caspofungin) and a non-echinocandin inhibitor (ibrexafungerp) of beta-1,3-glucan synthase (GS) in the BG2 strain background. Contrary to the other antifungals, caspofungin susceptibility exhibited high dependence on sphingolipid genes and low dependence on mitochondrial genes. Numerous deficiencies in mitochondrial genes increased fitness in micafungin, anidulafungin, rezafungin, and ibrexafungerp largely through effects on Pdr1, a stress-activated transcription factor known to promote fluconazole resistance. Several targets of Pdr1 (*RTA1, LAF1, LAC1, IPT1,* and *RSB1*) altered resistance to one or more of the GS inhibitors when overexpressed individually. Though Gal11 and other subunits of the mediator complex were necessary for the Pdr1 effects, an inhibitor of Gal11 (iKIX1) did not synergize with micafungin due to off-target antagonistic effects. Substantial variation was observed in the genetic resistance spectra for the different GS inhibitors within the BG2 strain background and comparison to the CBS138 strain background. The intra-strain variation was linked to gene deficiencies thought to alter phospholipid and ergosterol composition or asymmetry in cellular membranes. The findings generally support and extend earlier hypotheses that a lipid code governs GS inhibition and antifungal susceptibility in *C. glabrata*.

**IMPORTANCE:**

Echinocandins and ibrexafungerp are important antifungals that target the same fungal enzyme. When the fungus acquires resistance to one of these antifungals, it may or may not exhibit cross-resistance to the others. This study investigates how every gene in the pathogenic yeast *Candida glabrata* contributes to resistance and cross-resistance to all five antifungals of this type. It offers new insights into how each antifungal interacts with the target enzyme and identifies the antifungals where cross-resistance is common or rare, providing guidance on the sequences and combinations that may be most effective in clinical settings.

## INTRODUCTION

Fungal pathogens cause more than 1 billion serious infections each year that require medical intervention, a fraction of which leads to life-threatening diseases, especially among patients with compromised immune systems (1). Infection of the bloodstream and dissemination to vital organs by the *Candida* family of yeasts remains hard to treat, with mortality rates ranging from 30% to 60% (2–6). Of the causative species, *Candida albicans* is responsible for the majority of infections. Though *Candida glabrata* (also known as *Nakaseomyces glabratus*) is the second most common cause of candidiasis, its prevalence has been rising rapidly, possibly due to its innate resistance to azole-class antifungals and easily acquired resistance to echinocandin-class antifungals (7–9). *C. glabrata* and *Candida auris* isolates often exhibit resistance to both classes of antifungals, while a few isolates of *C. auris* exhibit pan-resistance even to a third class of antifungals, the polyenes (10–14). While new classes of antifungals will be required to mitigate emerging fungal threats, a better understanding of how the existing antifungals work and how fungal pathogens respond to them could lead to improved therapeutic strategies.

Clinical resistance to echinocandins typically arises through gain-of-function (GOF) mutations in the target genes (*FKS1* and *FKS2*), which redundantly encode catalytic subunits of beta-1,3-glucan synthase (GS), an enzyme responsible for synthesizing a critical component of the cell wall (15). The echinocandin-resistance mutations most commonly change codons within two different “hot-spot” regions of *FKS1* and *FKS2* (16), which cluster close together in the experimentally determined three-dimensional structures of the Fks1 protein of *Saccharomyces cerevisiae* (17). Most amino acid substitutions confer cross-resistance to all three of the original echinocandins: caspofungin, micafungin, and anidulafungin (12, 18). However, several different GOF substitutions in Fks1 were found to differentiate caspofungin from the other two echinocandins (19), suggesting the binding sites of these antifungals only partially overlap. Deep mutational scanning of the hot-spot regions of Fks1 and Fks2 in *S. cerevisiae* provides additional details of pan-drug and drug-specific resistance to echinocandins (20). Recently, a new long-lived echinocandin related to anidulafungin (rezafungin) was approved for clinical use (21). Additionally, a novel non-echinocandin class of GS inhibitor that is structurally different from the echinocandins (ibrexafungerp) was recently approved for the treatment of vulvovaginal candidiasis (22). Some of the GOF mutants that were strongly resistant to all echinocandins remained susceptible to ibrexafungerp and vice versa (23). The findings predict partially overlapping binding sites of all the GS inhibitors, including ibrexafungerp.

Apart from the GOF mutations in GS itself, many patient isolates and laboratory isolates harbor loss-of-function (LOF) mutations in other genes that confer significant resistance to inhibitors (24, 25). LOF mutations are not commonly observed in diploid fungi such as *C. albicans* and *Candida parapsilosis* because the function of the mutated gene is usually complemented by the unmutated copy. However, LOF resistance mutations are expected to arise at high rates in haploid species such as *C. glabrata* and *S. cerevisiae* and may contribute substantially to clinical outcomes if the effect size outweighs the fitness defect. LOF mutations in the mitochondrial genome (*rho-, rho0*) or many nuclear genes encoding mitochondrial proteins (*petite*) have been found to increase resistance of *C. glabrata* to micafungin (26). The molecular mechanism underlying this effect may include the activation of Pdr1, a stress-responsive transcription factor whose downstream targets, including *RTA1*, were recently shown to influence micafungin resistance (27). The same group of mitochondrial LOF mutations was previously shown to activate Pdr1 and induce the expression of another target, *CDR1*, which encodes a lipid flippase that effluxes fluconazole from the fungal cell, producing azole resistance (28). A variety of *GOF* mutants of *PDR1* have been isolated from azole-treated patients that appear to bypass the requirement for upstream signals that emanate from dysfunctional mitochondria (29). *PDR1-GOF* mutants may have smaller fitness defects in host niches and even promote fitness in the bloodstream (30), unlike mitochondrial LOF mutants.

Interestingly, LOF mutations in several genes required for sphingolipid biosynthesis have been shown to increase resistance to caspofungin, while at the same time decreasing resistance to micafungin in *C. glabrata*, *C. albicans*, and the mold *Aspergillus nidulans* (31, 32). LOF mutations in *FEN1*, which encodes an enzyme functioning early in sphingolipid biosynthesis, have been found in *C. glabrata* isolates from caspofungin-treated patients (25). These findings suggest that sphingolipids somehow potentiate the interactions between caspofungin and GS and depress the interactions between micafungin and GS. It remains unclear whether the other approved GS inhibitors behave like caspofungin or micafungin in their dependence on sphingolipids or whether other types of lipids also contribute to the inhibition of GS. If such discrimination holds for all the different GS inhibitors, it may be possible to achieve better clinical outcomes simply by treating patients with GS inhibitors in an optimal combination or sequence.

This study utilizes the *Hermes* transposon to generate a vast spectrum of LOF mutations in *C. glabrata* and quantifies their relative fitness when grown at different doses of all four approved echinocandins and ibrexafungerp. It also profiles LOF mutations in isogenic *pdr1∆* and *PDR1-GOF* derivatives of BG2, a vaginal isolate, and compares the findings to those obtained recently in the fecal isolate CBS138 that exhibits large (~1%) sequence divergence (27). We provide many new insights into the roles of mitochondria, Pdr1, and lipids in resistance to GS inhibitors, as well as identify LOF mutations that increase echinocandin susceptibility *in vitro*.

## RESULTS

### Micafungin resistance genes in BG2 overlap with those in CBS138

**Fig 1:**
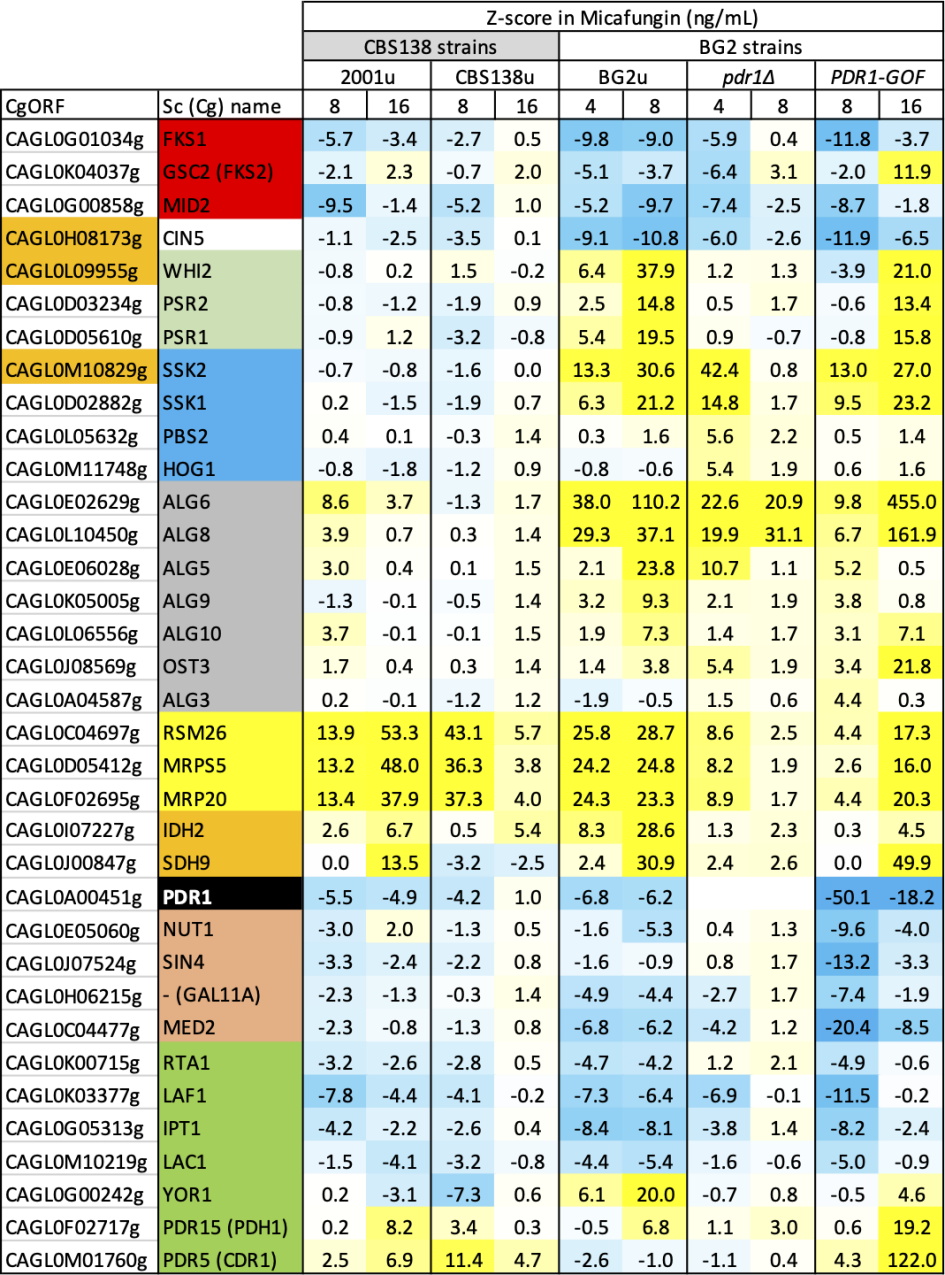
Micafungin resistance genes overlap in BG2 and CBS138 strains. Pools of transposon insertion mutants in strains BG2u, *pdr1∆*, and *PDR1-GOF* were grown in several concentrations of micafungin and subjected to Tn-seq analyses. The *Z*-scores for 27 genes of interest were tabulated, colorized on a scale from strongly depleted (blue) to strongly enriched (yellow), and compared to *Z*-scores obtained from a previous study using the CBS138-derived strains CBS138u and 2001u (27). Three ORFs known to be mutated in CBS138 and functional in BG2 are indicated (orange). Genes that function together in various processes of *S. cerevisiae* are sorted and colorized.

**Fig 2:**
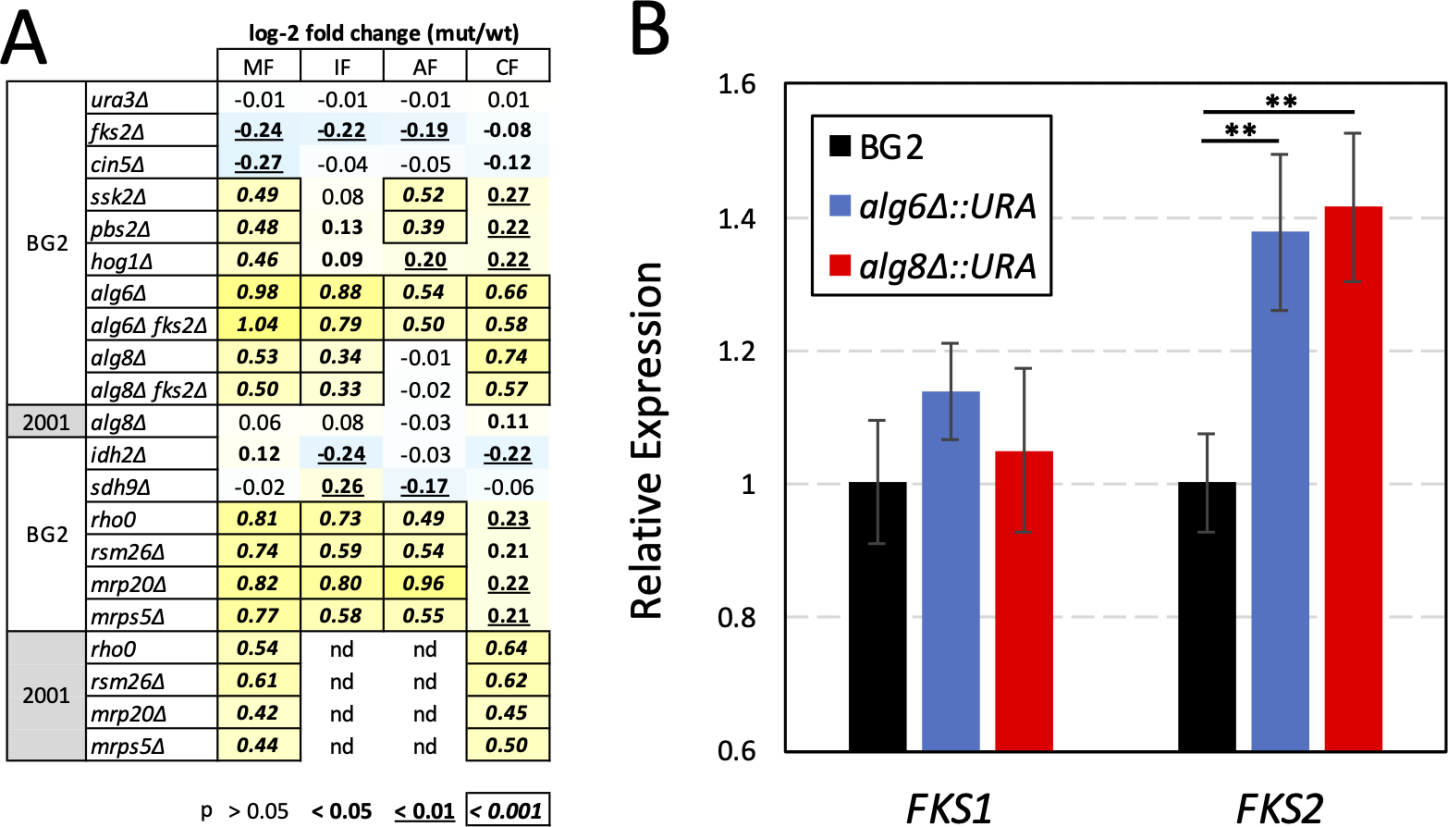
Quantification of antifungal resistance exhibited by individual gene knockout mutants and endogenous gene expression. (**A**) The indicated genes were knocked out in strains BG2u or 2001u, and the resulting mutants were grown in varying concentrations of micafungin (MF), ibrexafungerp (IF), anidulafungin (AF), or caspofungin (CF) to determine the concentrations that cause a 50% reduction in maximal growth (IC50s). Four replicate IC50s were averaged, ratioed to that of the wild-type parent strain, transformed to log scale (numbers), tabulated, and colorized from lowest (blue) to highest (yellow). Student’s *t*-test was used to calculate *P*-values, and three levels of significance are indicated (bold, underscore, and box). (**B**) Expression of *FKS1* and *FKS2* genes was measured by qRT-PCR in exponentially growing cultures of the indicated strains.

Genes that alter susceptibility to several different doses of micafungin were identified by performing Tn-seq analyses on a complex pool of transposon insertion mutants in BG2u, a *ura3∆* derivative of strain BG2, using a protocol identical to that used previously for strains CBS138u and 2001u, which are *ura3∆* derivatives of strain CBS138 that, respectively, lack and contain a segmental duplication spanning 60 genes (27). As before, the *Z*-scores were calculated for each annotated gene in each condition to identify genes that are significantly enriched or depleted in the pool. Most genes did not appreciably alter relative fitness at any dose of micafungin (|*Z*| < 3.0; Table S1). The *FKS1* and *FKS2* genes, which encode the catalytic subunits of GS, and *MID2*, which encodes a positive regulator of Rho1, an essential subunit of GS (33–35), were significantly underrepresented (*Z* < −3.0) at multiple doses in the BG2u pool, as observed previously in CBS138u and 2001u (Fig. 1). Overall, the *Z*-scores obtained for BG2u at 4 ng/mL micafungin correlated best with 8 and 16 ng/mL for strains CBS138u and 2001u, with high Pearson correlation coefficients (PCC = 0.69 and 0.72, respectively; Table S2). Three genes that are non-functional in CBS138 due to premature stop codons (Fig. 1; orange gene names) exhibited prominent *Z*-scores in BG2u but not in CBS138u. One such gene was *CIN5* (*Z* = −10.8), which encodes a repressor of mitochondrial functions (28). In broth microdilution assays, a *cin5∆* mutant of BG2u exhibited an IC50 for micafungin that was significantly lower than the wild-type control (log_2_[fold change] =−0.27; Fig. 2A). Another was *WHI2*, which encodes a regulatory subunit of the protein phosphatases encoded by *PSR1* and *PSR2* (36), all of which exhibited high *Z*-scores in micafungin (Fig. 1). The third gene that was non-functional in CBS138u but functional and impactful in BG2u was *SSK2*, which encodes a MAPKKK of the high osmolarity (HOG) signaling pathway (37). The *SSK1* gene that functions upstream of *SSK2* also exhibited high *Z*-scores in BG2u, while two genes that function downstream of *SSK2* (*PBS2* and *HOG1*) exhibited near neutral *Z*-scores (Fig. 1). Nevertheless, the *pbs2∆* and *hog1∆* knockout mutants in BG2u exhibited significantly increased resistance to micafungin similar to that of the *ssk2∆* mutant (Fig. 2A). These findings suggest that the already low level of strain-to-strain variation in micafungin resistance can be partially attributed to several gene losses that occurred uniquely in ancestors of CBS138.

The gene exhibiting the largest difference in *Z*-scores between BG2u and CBS138u was *ALG6* (Fig. 1). Several other genes involved in asparagine-linked glycosylation, such as *ALG8*, *ALG5*, *ALG9*, *ALG10*, and *OST3*, also exhibited increased micafungin fitness in BG2u but not CBS138u when disrupted with transposons (Fig. 1). Gene knockout mutants of *ALG6* and *ALG8* in BG2u resulted in significantly elevated resistance to micafungin in contrast to the very small effect of the *alg8∆* knockout mutation in 2001u (Fig. 2A), confirming the strain difference observed by Tn-seq. The *alg6∆* and *alg8∆* mutants of BG2u have been shown to cause chronic stresses that weakly activate calcineurin signaling and increase the expression of target genes such as *RCN2* (38). The expression of the *FKS2* gene was increased weakly (1.4-fold) but significantly (*P* < 0.01) in the *alg6∆* and *alg8∆* mutants relative to BG2u, as quantified by qRT-PCR in reference to control genes, while *FKS1* expression remained constant (Fig. 2B). Unexpectedly, the elevated micafungin resistance of the *alg6∆* and *alg8∆* mutants was not diminished upon introduction of the *fks2∆* mutation in contrast to BG2u (Fig. 2A). These findings suggest that genetic deficiencies in N-glycosylation increase resistance to micafungin through unknown *FKS2*-independent processes that are stronger in strains derived from BG2 than CBS138.

### Mitochondrial dysfunction and Pdr1 promote resistance to micafungin in *C. glabrata* strains

**Fig 3:**
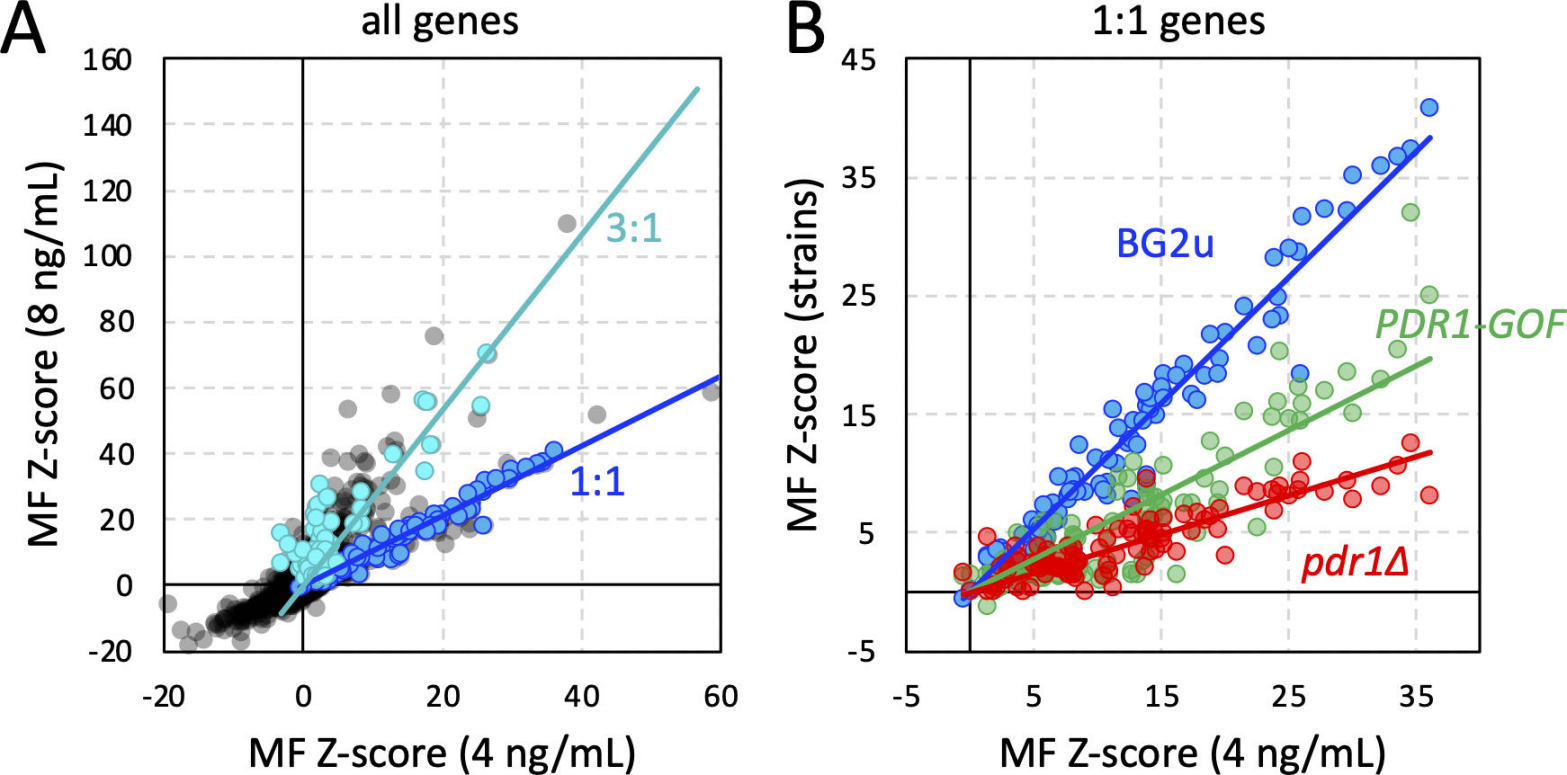
*Z*-score comparisons between different doses of micafungin and different BG2u-derived strains. (**A**) *Z*-scores from all genes in BG2u obtained at 4 and 8 ng/mL micafungin were charted (gray symbols). The 1:1 subgroup (blue symbols) and the 3:1 subgroup (green symbols) of mitochondrial genes are highlighted. (**B**) Micafungin *Z*-scores for 101 mitochondrial genes in the 1:1 subgroup were obtained from BG2u (8 ng/mL), *pdr1∆* (4 ng/mL), and *PDR1-GOF* (16 ng/mL) strains and charted against those from BG2u (4 ng/mL) as indicated. *R*^2^ correlation coefficients ranged from 0.89 to 0.98.

Hundreds of genes encoding mitochondrial proteins have been found to confer significant resistance to micafungin when disrupted with transposons in CBS138-derived strains (27). In strain BG2u, 201 mitochondrial gene deficiencies exhibited significantly increased fitness in micafungin (*Z* > 3.0) at 4 or 8 ng/mL micafungin (Table S1). Interestingly, when the *Z*-scores obtained at these two doses were plotted against each other (Fig. 3A), the mitochondrial genes separated into two groups: one that exhibited similar *Z*-scores at both doses (1:1 subgroup) and another that exhibited roughly threefold higher *Z*-scores at 8 ng/mL relative to 4 ng/mL (3:1 subgroup). The 1:1 subgroup (blue symbols) contained numerous genes required for translation of mitochondrial mRNAs, including nearly all genes encoding subunits of the mitoribosome, as well as tRNA synthetases and initiation, elongation, and termination factors (101 genes). The 3:1 subgroup (cyan symbols) contained genes that were highly enriched in the mitochondrial TCA cycle and electron transport chain, many of which were not significant in CBS138u or 2001u at any dose of micafungin (Table S1). Knockout mutants from the 1:1 group (*rsm26∆, mrps5∆*, and *mrp20∆*) and the 3:1 group (*idh2∆* and *sdh9∆*) were generated in BG2u and assayed for resistance to micafungin using the broth microdilution method. Knockout mutants from the 3:1 group exhibited little or no increased resistance to micafungin (0.99- to 1.1-fold; converted to log_2_-fold change in Fig. 2A). Knockout mutants lacking the 1:1 genes exhibited approximately 1.7-fold increased resistance to micafungin similar to a *rho0* strain lacking the mitochondrial genome (Fig. 2A). These findings generally validate the Tn-seq findings and unexpectedly split the mitochondrial genes into two functional groups with detectably different contributions to micafungin resistance.

Mitochondrial deficiencies potently activate the Pdr1 transcription factor and induce the expression of several target genes such as *CDR1* (39–41). As expected, the *PDR1* gene and several of its targets exhibited significantly negative *Z*-scores at two middle doses of micafungin (Fig. 1). To explore these effects more broadly, pools of transposon insertion mutants were generated in *pdr1∆* mutants and *PDR1-GOF* (gain-of-function) derivatives of BG2u and analyzed by Tn-seq as before (27). As expected by altered sensitivity, the *pdr1∆* and *PDR1-GOF* pools exhibited the most outlier genes at lower (4 ng/mL) and higher (16 ng/mL) doses of micafungin, respectively, relative to BG2u (8 ng/mL). At these doses, *FKS1, FKS2, CIN5, SSK2, ALG6*, and related genes were all significant outliers in both the *pdr1∆* and *PDR1-GOF* strains (Fig. 1), suggesting they regulate micafungin resistance independent of Pdr1. In contrast to these genes, mitochondrial genes in both the 1:1 and 3:1 subgroups exhibited much lower *Z*-scores in *pdr1∆* and *PDR1-GOF* mutants than in the wild-type BG2u strain. When *Z*-scores of the 1:1 subgroup from BG2u (101 genes) were plotted against those of *pdr1∆* and *PDR1-GOF* mutants at these doses (Fig. 3B), strong correlations were observed (*R*^2^ = 0.92 and 0.89, respectively), indicating very high reproducibility between the independently generated pools. However, the slopes were 3.3- and 1.9-fold lower than BG2u, respectively. These findings show that mutants causing mitochondrial dysfunction increased fitness in micafungin mostly through Pdr1-dependent effects but also through Pdr1-independent effects.

**Fig 4:**
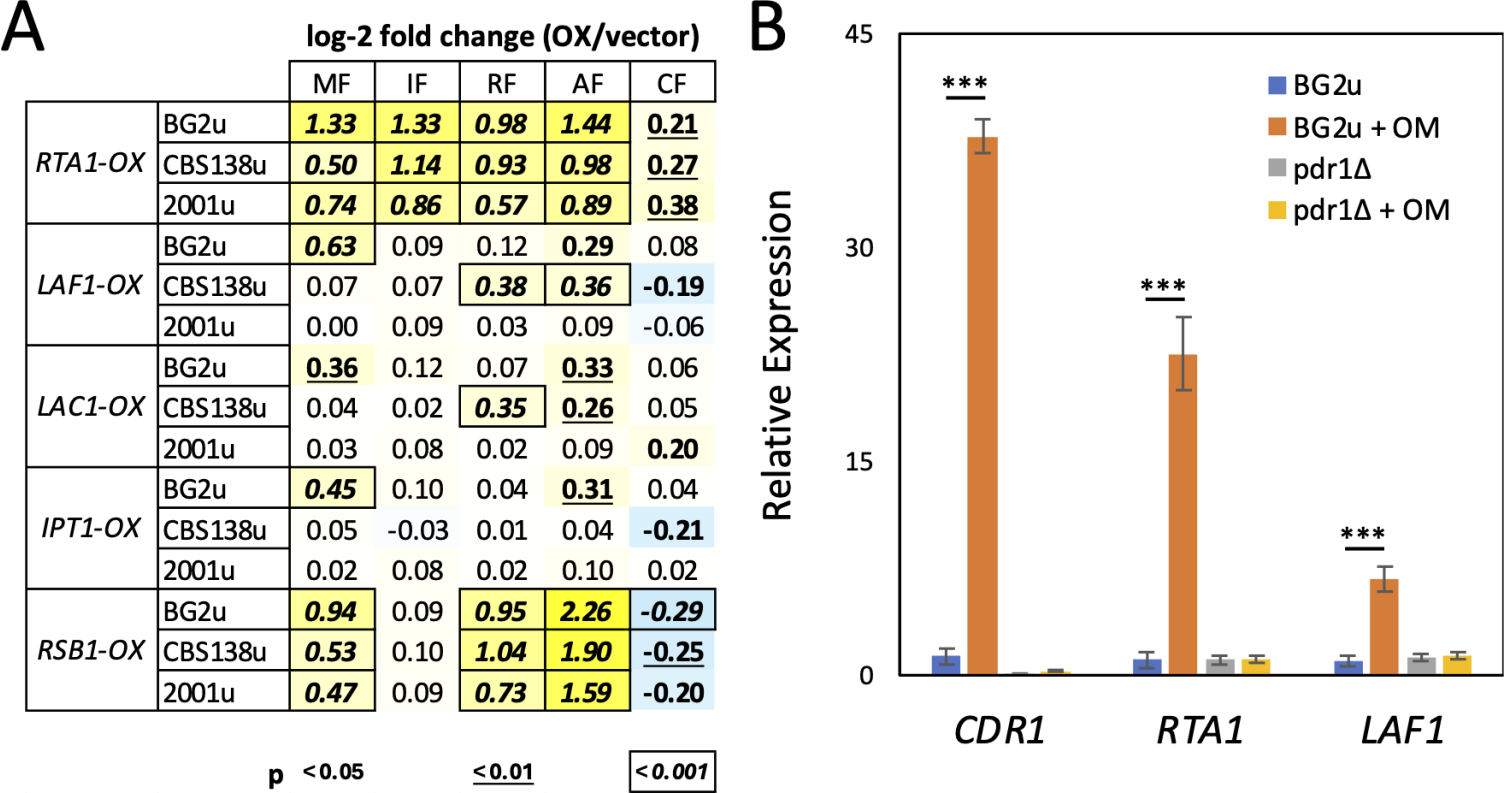
Quantification of antifungal resistance exhibited by individually overexpressed genes and endogenous gene expression. (**A**) Centromeric plasmids bearing the indicated genes under the control of a strong constitutive promoter were transformed into the indicated strains and assayed by broth microdilution for changes in antifungal resistance relative to the empty parent plasmid (pCN-PDC1). Log-2-fold changes (numbers) were colorized and tested for statistical significance as described in Fig. 2. (**B**) Expression of endogenous *CDR1*, *RTA1*, and *LAF1* genes was measured by qRT-PCR in strains BG2u and *pdr1∆* that have been exposed to oligomycin (+OM) for 1 h. Bars indicate averages of four replicates (±SD), and *** indicates *P* < 0.001 in a Student’s *t*-test.

Dozens of genes have been identified as direct targets of the Pdr1 transcription factor (42–45). Among that set, four genes (*RTA1*, *LAF1*, *IPT1*, and *LAC1*) exhibited *Z*-scores less than −3.0 at the two middle doses of micafungin in both strain backgrounds (Fig. 1; Table S1). Two others (*PDH1* and *CDR1*) exhibited *Z*-scores greater than +3.0 in both CBS138-derived strains as well as the *PDR1-GOF* derivative of BG2u (Fig. 1; Table S1). One other target of Pdr1 (*YOR1*) exhibited negative *Z*-scores in CBS138 and positive *Z*-scores in BG2 strains. The opposing behaviors of these Pdr1 targets suggest complexity in how Pdr1 confers micafungin resistance. The coding sequences of *RTA1, LAF1, IPT1,* and *LAC1* genes were all cloned into a centromeric expression plasmid downstream of the strong promoter of *PDC1* (46) and introduced into BG2u, CBS138u, and 2001u strains. *RTA1* overexpression produced significant micafungin resistance in all three strains, whereas *LAC1, LAF1,* and *IPT1* overexpression produced micafungin resistance in only the BG2u strain (Fig. 4A). The extra copy of the *RTA1* gene in the 2001u strain that bears a segmental duplication also increased resistance to micafungin (27). To confirm that Pdr1 activation increases the expression of *RTA1* and *LAF1*, mRNA levels were quantified by qRT-PCR after BG2u cells were exposed to the mitochondrial respiration inhibitor oligomycin. Expression levels of *RTA1* and *LAF1* increased by 23- and 7-fold, respectively, in a Pdr1-dependent way similar to *CDR1* (Fig. 4B). Therefore, the mitochondria-Pdr1-Rta1 pathway of micafungin resistance appeared to be conserved in BG2 and CBS138 strains of *C. glabrata*, while other targets of Pdr1 produced weaker strain-specific effects that may also antagonize one another, thus diminishing the net effects of Pdr1.

### Ibrexafungerp resistance mechanisms in BG2u

Ibrexafungerp is an orally available inhibitor of fungal GS that is chemically unrelated to micafungin and the other echinocandins (22). Overexpression of *RTA1* increased resistance to ibrexafungerp to about the same degree as micafungin in all three strains tested, while overexpression of *LAF1*, *LAC1*, and *IPT1* had no impact on resistance to ibrexafungerp (Fig. 4A). To explore genetic resistance to ibrexafungerp, Tn-seq screens were performed on the same pools of insertion mutants and conditions employed above for micafungin. Outlier genes were observed mostly at one dose (32 ng/mL) for *pdr1∆* mutants and two doses (32 and 64 ng/mL) for BG2u. Like micafungin, the two doses of ibrexafungerp resolved mitochondrial genes into 1:1 and 3:1 subgroups, both of which depended partially on Pdr1 (Table S1). These findings suggest that mitochondrial deficiencies can increase fitness in ibrexafungerp through Pdr1-dependent and Pdr1-independent effects similar to micafungin. Overall, the genes that impact fitness in ibrexafungerp were highly correlated to those impacting fitness in micafungin at two middle doses of each (PCC = 0.82 and 0.76; Table S2).

**Fig 5:**
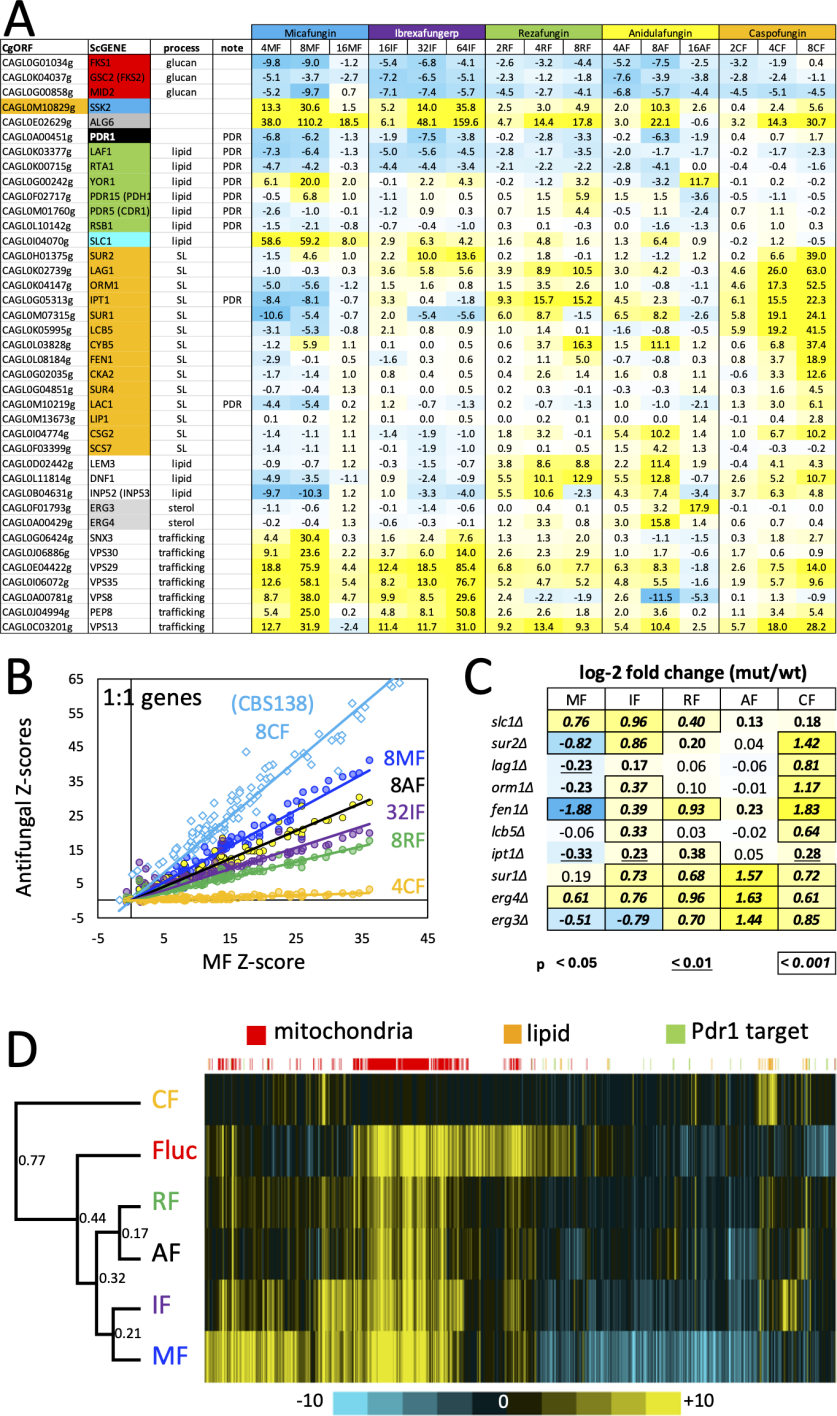
Comparisons of GS inhibitors. (**A**) *Z*-scores for selected genes of BG2u were tabulated from multiple doses of the five GS inhibitors and colorized as described in Fig. 1. (**B**) *Z*-scores obtained for the 1:1 subgroup of mitochondrial genes in strain BG2u (circles) calculated after growth in 8 ng/mL micafungin (MF; blue), 32 ng/mL ibrexafungerp (IF; purple), 8 ng/mL rezafungin (RF; green), 8 ng/mL anidulafungin (AF, yellow), and 4 ng/mL caspofungin (CF, orange) were charted against those obtained in micafungin (4 ng/mL). The same genes in strain 2001u (diamonds) exposed to 16 ng/mL micafungin were charted against those obtained in 8 ng/mL caspofungin (MF; turquoise diamonds). All trendlines were highly correlated (*R*^2^ between 0.79 and 0.98). (**C**) Resistance of individual gene knockout mutants in BG2u to the five different GS inhibitors was quantified and visualized as described in Fig. 2A. (**D**) Hierarchical clustering was performed on antifungals and antifungal resistance genes in strain BG2u based on average *Z*-scores from two doses. Relatedness of antifungals was depicted on the left with branch lengths (numbers) equal to 1 – PCC. Mitochondrial genes (red bars) and lipid genes (orange) were frequently clustered together. Scale bar indicates the average *Z*-scores of each gene.

Several genes stood out as differentially impacting resistance to ibrexafungerp relative to micafungin (Fig. S1). For example, *SLC1* encoding an enzyme involved in phospholipid biosynthesis exhibited a much higher *Z*-score in micafungin versus ibrexafungerp (*Z* = 59 versus 6.3), while several genes involved in sphingolipid biosynthesis (*SUR2, LAG1*, *ORM1, FEN1, LCB5, IPT1,* and *CKA2*) exhibited the reverse behavior (Fig. 5A), particularly in the *pdr1∆* mutant background (Table S1). Knockout mutants of these genes largely corroborated the Tn-seq findings, except for *slc1∆,* which had a similar resistance to both antifungals in monocultures (Fig. 5C).

### Rezafungin and anidulafungin resistance mechanisms in BG2u

Rezafungin and anidulafungin are echinocandins related to micafungin. Tn-seq analyses of these antifungals were performed on the BG2u insertion pool in the same manner as described above. As expected, the genes encoding GS (*FKS1* and *FKS2*) were significantly underrepresented at several different doses of both antifungals. *PDR1* and *RTA1* also exhibited significantly negative *Z*-scores in both antifungals (Fig. 5A; Table S1). Overall, the correlation between rezafungin, anidulafungin, micafungin, and ibrexafungerp was high (PCC ranging from 0.76 to 0.84; Table S2). The 1:1 and 3:1 subgroups of mitochondrial genes again exhibited positive *Z*-scores that were distinguishable from each other at different doses of rezafungin and anidulafungin (Table S1). However, some genes involved in lipid metabolism (e.g., *LEM3, DNF1, INP52, CYB5, SUR1, CSG2,* and *IPT1*) exhibited much stronger fitness effects in these two echinocandins relative to micafungin and ibrexafungerp, while genes involved in membrane trafficking (e.g., *VPS29, VPS35, VPS8,* and *PEP8*) exhibited much weaker effects (Fig. 5A; Fig. S1). Insertions in the *ERG3* and *ERG4* genes, which function in ergosterol biosynthesis, increased fitness in anidulafungin only (*Z* = 17.9 and 15.8; Fig. 5A). LOF mutations in *ERG3* were previously associated with anidulafungin resistance in long-term evolution experiments (10). Knockout of these genes in BG2u increased resistance to anidulafungin more than rezafungin (Fig. 5C). Overexpression of the lipid flippase gene *RTA1* conferred resistance to all the GS inhibitors in both strain backgrounds (Fig. 4A). Interestingly, overexpression of a related lipid flippase encoded by *RSB1*, which is not a target of Pdr1 (47), resulted in strong resistance to anidulafungin, moderate resistance to rezafungin and micafungin, and no resistance to ibrexafungerp (Fig. 4A). The drug-specific phenotypes of genes involved in membrane asymmetry, trafficking, and biosynthesis of lipids suggest that each GS inhibitor prefers a unique lipid composition for maximum efficacy against the targets. This conclusion is reinforced below by studies of caspofungin.

### Caspofungin resistance mechanisms

Interestingly, the overexpressed *RTA1* gene caused a much milder increase in caspofungin resistance compared to the other inhibitors, while the overexpressed *RSB1* gene decreased caspofungin resistance similarly in all three strains tested (Fig. 4A). *LAF1, LAC1,* and *IPT1* overexpression had very small and inconsistent effects. These findings suggest that *PDR1* may have little or no impact on caspofungin resistance in BG2u. This prediction was supported by Tn-seq analysis over a broad range of caspofungin doses (Fig. 5A; Table S1). Neither *PDR1* nor any of its targets exhibited decreased fitness in caspofungin, while only *LAC1* and *IPT1* exhibited increased fitness in caspofungin when disrupted with transposons (Fig. 5A; Table S1). Furthermore, the 1:1 subgroup of mitochondrial genes exhibited approximately 20-fold lower *Z*-scores in caspofungin relative to micafungin, with only one gene producing a *Z*-score greater than 3.0 (Fig. 5B). As controls, the *ALG6* and *SSK2* genes exhibited strongly positive *Z*-scores, while the *FKS1, FKS2,* and *MID2* genes exhibited negative *Z*-scores at multiple doses of caspofungin (Fig. 5A; Fig. S1). Because hundreds of mitochondrial genes and other genes were not significantly enriched or depleted in caspofungin, the overall correlation between caspofungin and the four other GS inhibitors was very low (PCC = 0.52, 0.32, 0.21, and 0.33). To visualize the relationships between all the antifungals, hierarchical clustering was performed using the average *Z*-scores from two middle doses of each drug treatment. Remarkably, caspofungin was a distant outlier from the other drugs (Fig. 5D). Even fluconazole clustered closer to micafungin, ibrexafungerp, anidulafungin, and rezafungin than did caspofungin, largely because of the differential impact of mitochondrial genes.

To test whether CBS138-derived strains exhibit caspofungin resistance spectra similar to BG2u, Tn-seq analysis was performed on the pool of insertion mutants in strain 2001u. Remarkably, 148 mitochondrial genes exhibited *Z*-scores greater than 3.0 (Table S1), and the *Z*-scores from the 1:1 subgroup were well-correlated with those of micafungin (Fig. 5B). We explored the strain variation more deeply using *rho0* mutants and three knockout mutants of the 1:1 subgroup in broth microdilution assays. These mutants all produced stronger resistance to caspofungin in the 2001u background than in the BG2u background (Fig. 2A). Overall, the caspofungin and micafungin resistance spectra were correlated much better in this CBS138-derived strain (PCC = 0.66) than in the BG2u strain (PCC = 0.21).

**Fig 6:**
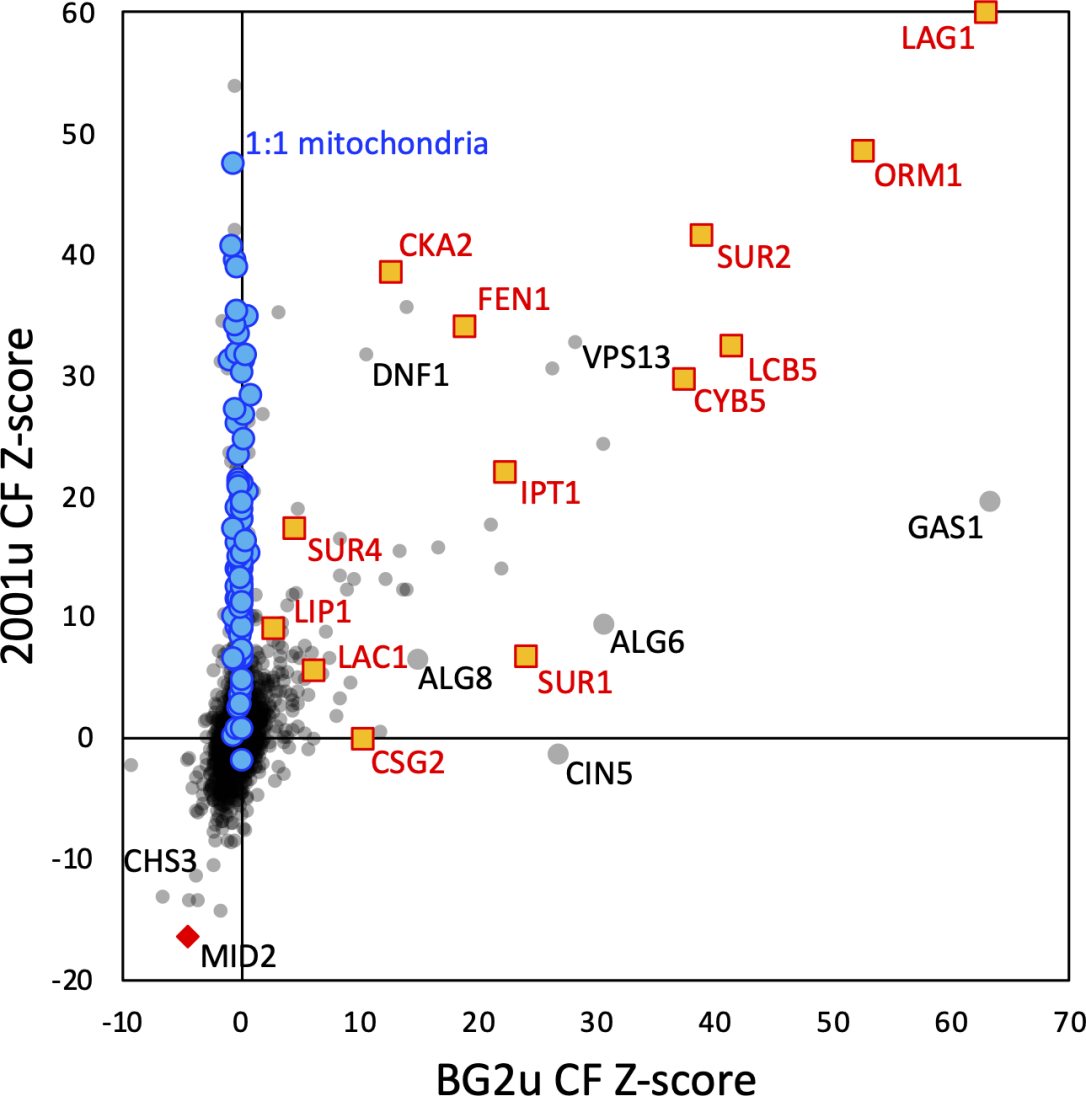
BG2u and 2001u differ in caspofungin resistance spectra. Caspofungin *Z*-scores obtained from 2001u at 8 ng/mL were charted against those from BG2u (gray circles). Mitochondrial genes (1:1, blue circles) and sphingolipid genes (orange squares and red names) are highlighted along with several other genes.

When *Z*-scores from both BG2u and 2001u strain backgrounds were compared (Fig. 6), caspofungin susceptibility was strikingly dependent on a set of 11 genes that function at different steps in the sphingolipid biosynthesis pathway. Two additional sphingolipid genes were significant in one strain or the other. Of these 13 sphingolipid genes, only two to five genes had significantly positive *Z*-scores in the other four GS inhibitors that were generally smaller (Table S1). Five of the sphingolipid genes (*SUR1, IPT1, ORM1, LAC1,* and *LCB5*) exhibited strongly negative *Z*-scores in micafungin in striking contrast to caspofungin (Fig. 5A; Table S1). Consistent with all these *Z*-scores, knockout mutants of seven sphingolipid genes in BG2u exhibited significantly increased resistance to caspofungin, several of which exhibited decreased resistance to micafungin (Fig. 5C). Previous studies in strain 2001u showed that knockout mutants of two sphingolipid genes (*FEN1* and *CKA2*) increased resistance to caspofungin and decreased resistance to micafungin (32). Altogether, the findings generally support and extend the earlier hypothesis that sphingolipids facilitate caspofungin inhibition of GS and other lipids facilitate inhibition by micafungin (32).

### Minimizing resistance to antifungals in *C. glabrata*

Genes that exhibit consistently negative *Z*-scores across different antifungals and strains could be targeted for the development of new co-drugs that may augment the potency of existing antifungals. To identify such genes, each gene was indexed to the average *Z*-scores of *FKS1, FKS2,* and *MID2* across all conditions, including fluconazole, but excluding *pdr1∆* and *PDR1-GOF* data sets (see Materials and Methods). Twelve genes involved in the biosynthesis of mannan (*YND1, KRE2, PMT2, VAN1, MNN2, GDA1, MNS1,* and *MNN11*) and chitin (*CHS3-B, CHS6, CHS7,* and *PFA4*) components of the cell wall were among the functional groups with top-scoring genes. An inhibitor of mannan synthesis (SDZ 90-215) was previously found to exhibit additivity with micafungin *in vitro* (27). An inhibitor of chitin synthesis (nikkomycin Z) increased micafungin activity *in vitro* (48) and increased caspofungin activity *in vivo* (49).

**Fig 7:**
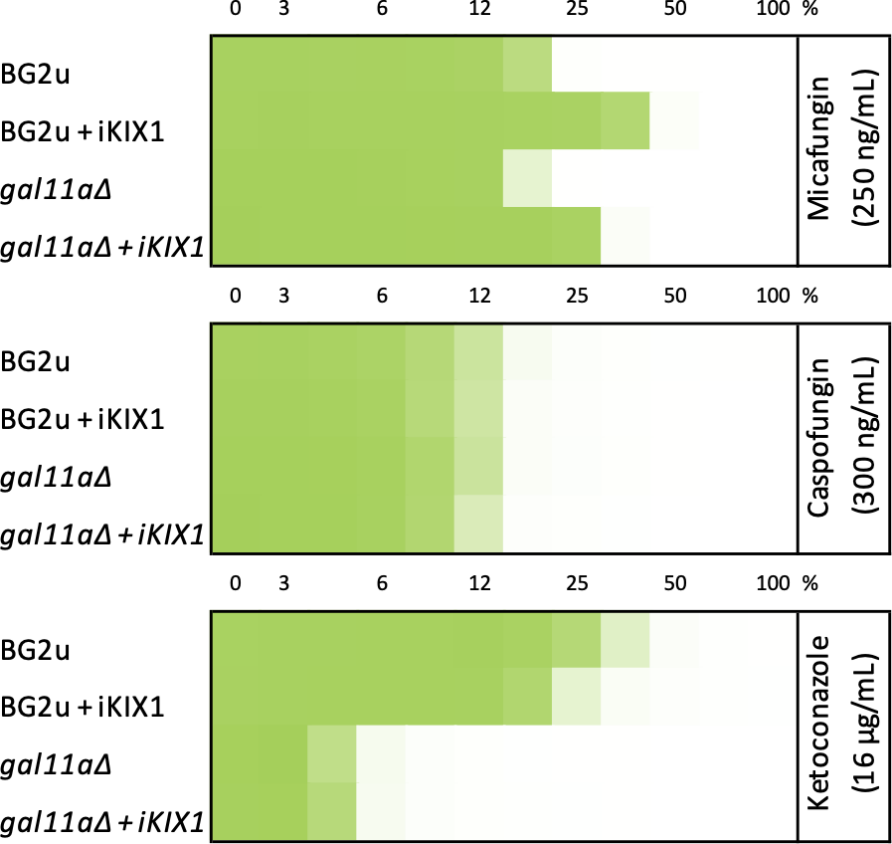
iKIX1 antagonizes micafungin independent of its target. Replicate cultures of BG2u and isogenic *gal11a∆* knockout mutants were incubated in SCD medium containing or lacking iKIX1 (10 µg/mL) in 96-well dishes containing varying concentrations of micafungin, caspofungin, or ketoconazole. After 20 h, growth was measured at 600 nm. Four replicates were averaged, charted as a function of drug dose (percentage of maximum indicated), and colorized on a scale of saturated growth (green) to no growth (white).

Interestingly, four subunits of the mediator complex (*MED2, GAL11A, SIN4,* and *NUT1*), which promote transcription of *CDR1* by interacting with Pdr1 (50), were also highly indexed. These genes exhibited much lower *Z*-scores in the *PDR1-GOF* derivative of BG2u than the *pdr1∆* mutant at all concentrations of micafungin (Fig. 1). A small molecule, termed iKIX1, has been found to bind Gal11a and interfere with Pdr1-dependent expression of *CDR1*, resulting in synergism with azoles (51). We tested whether iKIX1 can augment the potencies of micafungin and caspofungin as seen previously with the azoles. A moderate dose of iKIX1 (10 µg/mL) that increased the potency of ketoconazole on BG2 had no effect on caspofungin potency but strongly decreased the potency of micafungin (Fig. 7). A knockout mutant of the mediator complex (*gal11a∆*) lost the synergism between iKIX1 and ketoconazole but retained the antagonism between iKIX1 and micafungin (Fig. 7). These findings suggest that iKIX1 has off-target effects that selectively antagonize micafungin efficacy in addition to its known effects on Pdr1-mediator interactions. Next-generation inhibitors of mediator (52, 53) may lack this off-target antagonism and synergize with multiple GS inhibitors in addition to azoles.

## DISCUSSION

The four echinocandins and ibrexafungerp all block GS, damage the cell wall, activate stress response pathways, and ultimately cause lysis of the plasma membrane and fungal cell death. While genetic resistance to these antifungals can be achieved through rare GOF mutations in the GS-encoding genes (*FKS1* and *FKS2*), mutations in other genes can have large impacts in clinical settings (25). LOF mutations that alter resistance to antifungals can shed light on the biological processes that fungal cells naturally utilize to sense stresses, mitigate damage, and alter influx, efflux, access, or metabolism of the antifungals themselves (54). Broad transposon mutagenesis of *C. glabrata* and mutant profiling with next-generation sequencing were implemented here to efficiently explore the landscape of loss-of-function mutations that alter competitive fitness during exposure to low doses of four different GS inhibitors. The analysis revealed unexpectedly large differences between caspofungin and the four other GS inhibitors (micafungin, rezafungin, anidulafungin, and ibrexafungerp) in the BG2 strain background, but much less so in the CBS138 background. This strain difference primarily involves the different impacts of mitochondrial deficiencies, which produced less caspofungin resistance in BG2u than in 2001u. Mitochondrial deficiencies are known to activate the Pdr1 transcription factor in both strain backgrounds. However, disruption and overexpression of *RTA1*, *RSB1*, and several other Pdr1 targets impacted caspofungin resistance to similar degrees in both strain backgrounds. One or more of the three genes that are naturally mutated in CBS138 and functional in BG2 (*CIN5*, *WHI2*, and *SSK2*) may contribute to the differential effects of mitochondrial deficiencies. All three genes, plus other components of their regulatory pathways, altered micafungin resistance when disrupted with transposons through unknown processes in BG2u, but not in 2001u or CBS138u.

Within BG2u, caspofungin also deviated from the other antifungals through its large dependence on nearly all non-essential genes involved in sphingolipid biosynthesis. Only two of these genes exhibited weakly positive *Z*-scores in micafungin, while five others exhibited strongly negative *Z*-scores. The other three GS inhibitors depended on only a few of the sphingolipid genes. Several other genes involved in lipid biosynthesis, trafficking, or asymmetry in the membrane also appeared to influence the activity of only a subset of the GS inhibitors. The *DNF1* gene, encoding a lipid flippase (55, 56), seemed to match the patterns of some of the sphingolipid genes. An opposite pattern was exhibited by the *SLC1* gene, whose product synthesizes the lipid phosphatidic acid (57). *ERG3* and *ERG4* disruptions that alter the biosynthesis of ergosterol (58, 59) increased fitness only in anidulafungin. Even the ABC transporters *CDR1* and its paralog *PDH1*, which are both induced by Pdr1 activation and strongly involved in azole resistance (43), exhibited elevated fitness in micafungin when disrupted with transposons. Overexpression of the *RSB1* gene, encoding a lipid flippase related to Rta1, strongly increased resistance to anidulafungin, moderately increased resistance to rezafungin and micafungin, and decreased resistance to caspofungin, while having minimal impact on ibrexafungerp resistance. Altogether, the genetic findings support a model where each GS inhibitor depends on a particular repertoire of lipids in the two leaflets of the plasma membrane for maximal efficacy against the GS enzymes.

Cryo-electron microscopy of purified Fks1 from *S. cerevisiae* has revealed its active site near the cytoplasmic face of the plasma membrane (17, 60), which suggests that the GS inhibitors may need to flip from the outer to the inner leaflet of the plasma membrane or be transported through the membrane (61) to access its binding sites. Interestingly, more than 12 lipids were bound to the transmembrane domain of Fks1 in an orderly fashion, suggesting they regulate enzymatic activity (17). Several GOF mutations that confer echinocandin and ibrexafungerp resistance map to these lipid-bound surfaces in Fks1. Our genetic findings add support to hypothesis 1 of Hu et al. (17), which postulates that GS inhibition involves specific drug-lipid interactions in addition to specific drug-target and lipid-target interactions that both may be impacted by GOF mutations (20). Future research may elucidate the details of this hypothetical “lipid code” for GS inhibition.

LOF mutations in three sphingolipid genes (*FEN1*, *SUR2,* and *SUR4*), which have been isolated from patients undergoing caspofungin treatment (25), were also found to have similar behaviors. Mitochondrial gene deficiencies (petite mutants) have been recovered from untreated patients (62) and patients undergoing treatment with fluconazole (63, 64) or caspofungin (65). Such deficiencies are often overlooked due to the very small colony or “petite” phenotype produced on diagnostic agar media. Direct testing has demonstrated lower fitness of these petite mutants in multiple host environments, such as colonization of the gastrointestinal tract, proliferation within phagosomes of macrophages, and invasion of major organs from bloodstream infections (66). Several studies have hypothesized that *C. glabrata* and related pathogens may transiently generate mitochondrial deficiencies through genetic or epigenetic mechanisms that later can be reversed (62, 67). Such mechanisms could result in the activation of Pdr1 and increased expression of *CDR1*, *RTA1*, and other targets that positively impact both fitness and antifungal resistance in various host environments. During long-term treatment with fluconazole, GOF mutations in *PDR1* often arise and dominate the population as the resistance benefits outweigh the small costs in fitness imposed by hyperactive Pdr1 (29). GOF mutations in Pdr1 have not been reported in isolates that confer clinical resistance to GS inhibitors, possibly because the effects of *RTA1* induction are relatively mild. Additional Tn-seq experiments in clinically resistant isolates could reveal new strategies for combating antifungal resistance in the host.

The Tn-seq method also revealed candidate targets for the development of antifungal co-drugs, which might augment the potency of existing antifungals by disrupting the common mechanisms of intrinsic resistance. Previously, this concept was utilized to test whether micafungin potency *in vitro* can be increased by inhibitors of Chs3 (nikkomycin), Vrg4 (SDZ 90-215), and Gwt1 (manogepix) (27, 48, 49). Here, we tested iKIX1, an inhibitor of Gal11A and Gal11B subunits of the mediator complex (51), for its ability to increase micafungin potency. Surprisingly, moderate doses of iKIX1 that increased the potency of ketoconazole decreased the potency of micafungin, while having no impact on caspofungin potency. The antagonistic effect was observed in *gal11A∆* mutants, indicating an off-target effect of iKIX1. Off-target antagonism between caspofungin and rotenone, an inhibitor of mitochondrial respiratory complex I that is absent in *C. glabrata* (68), has been observed previously (26). Though these off-target effects were not mapped to any specific processes, they could conceivably involve lipids specifically required for micafungin resistance and not caspofungin resistance, and vice versa. Second-generation inhibitors of Gal11A/B have now been developed (52, 53), which potentially may lack the off-target effect and potentiate micafungin in addition to fluconazole, even when clinical resistance has occurred through gain-of-function mutations in Pdr1.

Resistance is only one of several fungal mechanisms that could contribute to therapeutic failures. Heteroresistance, where only a small subpopulation of fungal cells reversibly acquires echinocandin resistance, was recently linked to micafungin failure in *C. parapsilosis* (69). Tolerance mechanisms extend the lifespans of cells exposed to lethal doses of antibiotics or antifungals and, therefore, may contribute to the persistence of infections beyond the treatment period and eventual relapse. Mitochondrial dysfunction was recently shown to increase the tolerance of *C. glabrata* to micafungin and caspofungin *in vitro*, and tolerance may be induced in cells that have been engulfed by macrophages (66). The genes that regulate tolerance to GS inhibitors have not yet been fully investigated. Persistence mechanisms that regulate the percentage of tolerant cells within susceptible populations represent another underexplored means by which fungal cells survive our antifungal assaults (66, 70). Tn-seq screens could shed new light on the genes that regulate tolerance to antifungals and the formation of persister cells in a wide range of conditions and species.

## MATERIALS AND METHODS

### Transposon insertion profiling (Tn-seq)

Pools of transposon insertion mutants in the 2001u, BG2u, and *pdr1∆* mutant derivative of BG2u were described previously (27, 28). A new pool of insertion mutants in the *PDR1-GOF* strain was generated similarly. First, the *ura3∆ pdr1∆::HYG* strain (CGM1094) was converted to *ura3∆ PDR1-GOF* (TJN88) by transformation with a PCR product obtained from plasmid pLS9 (71) that carries *PDR1-R376W-Y584C-P822L*, a derivative of *PDR1* bearing three gain-of-function mutations within the core regulatory domain. This strain was transformed with pCU-MET3-Hermes, and then transposition was induced in 40 replicate cultures (72). The replicate cultures were pooled, enriched in 5-FOA, and cryo-preserved for Tn-seq as before (27, 28). The complex pools of insertion mutants were grown to saturation in SCD medium and diluted 1:100 into 300 mL of SCD medium containing a range of antifungals at different doses and shaken at 30°C for 48 h. To monitor growth, OD_600nm_ readings were taken at various points (see Fig. S2). Micafungin and anidulafungin were obtained from Cayman Chemicals, caspofungin from SeleckChem, rezafungin from Thermo Fisher Scientific, and ibrexafungerp from Scynexis. DNA from drug-treated pools was extracted, enriched for transposon insertion sites, and sequenced as previously described (27). The Quick-DNA Fungal/Bacterial Miniprep Kit from Zymo Research (Cat. #D6005) and the Bioruptor Pico sonication device were used to extract and fragment the DNA from each sample. The NEBNext Ultra II DNA Library Prep Kit for Illumina (Cat. #E7645) was used to prepare the sequencing library by adding indexed splinkerette adapters and enriching for the HERMES insertion site via PCR. DNA was size selected using AMPure XP magnetic beads from Beckman Coulter (Cat. #A63881). Paired-end reads were sequenced using the MiSeq Reagent Kit v3 (2 × 75 bp; Illumina) with custom primers specific to the HERMES transposon and the splinkerette to ensure that all reads were from genomic DNA at the transposon insertion site.

FastQ files were demultiplexed according to their index using CutAdapt and mapped to the BG2 v1 genome (73) or CBS138 v2 genome (74) with Bowtie2 (75). High-quality mapped reads (*Q* ≥ 20 with no mismatches at position 1) were used to tabulate the number of insertions at each recovered genomic site. For all sites within each annotated gene, the total number of Tn-seq reads was tallied, and the top site was removed to avoid possible passenger effects (27). *Z*-scores were calculated as previously described (27). Briefly, the total number of sequence reads at all sites within the coding sequences of every annotated gene obtained in each condition was plotted against that of the untreated control condition, and the number of standard deviations the gene deviates from the local average of genes was computed. These *Z*-scores (Table S1) were then used in hierarchical clustering after averaging the *Z*-scores from the two middle doses and filtering out any genes that did not have significant *Z*-scores in at least one of the drugs tested (|*Z*| ≤ 3). Clustering was performed using the R package pheatmap (76) to cluster both the rows and columns by their Pearson’s correlation distance based on the centroid method in order to generate a heatmap (Fig. 5D). These data were also indexed by multiplying the number of instances each gene exhibited a *Z*-score less than −3 with the Pearson correlation coefficient obtained for each gene across all conditions (excluding *pdr1∆* and *PDR1-GOF* strains).

### Generation of knockout mutants

All strains of *C. glabrata* and primers used in this study are listed in Table S3. Strain 2001 is from Kitada et al. (77), CBS138 is from Dujon et al. (78), BG2 is from Cormack and Falkow (79), and *pdr1∆* is from Orta-Zavalza et al. (80). Knockout mutants were made using the PRODIGE technique (81), which involves amplifying the *Saccharomyces cerevisiae URA3* or *HIS3* genes from the pRS406 or pRS403 plasmids (82) with 60-nucleotide primer homology arms that correspond to the flanking regions of the target gene. The transformants were grown on SCD-uracil or -histidine plates, followed by PCR authentication of URA+ colonies. The *ScURA3* transgene was removed by a similar method in which the hygromycin resistance gene was amplified from the pHYG-AID plasmid (83) with overhangs corresponding to the first and last 60 nucleotides of the *ScURA3* gene. Strains lacking a functional mitochondrial genome (*rho0* mutants) were generated by growing cells to log phase, back-diluting to 0.1 OD_600nm_, adding 25 µg/mL ethidium bromide, growing overnight at 30°C while shaking, plating cell dilutions on YPD plates, and selecting for colonies that were unable to grow on the non-fermentable carbon source glycerol (YPG plates). The *rho0* mutants were confirmed by the absence of *COX1* DNA using PCR (84).

### Generation of overexpression plasmids

BG2u genomic DNA was used as a template for PCR amplification of the *RTA1* and *LAF1* coding sequences (with 5′ SpeI and 3′ SalI extensions), *LAC1* and *RSB1* coding sequences (with 5′ SpeI and 3′ XhoI extensions), and *IPT1* (with 5′ XbaI and 3′ SalI extensions). The products and the expression plasmid pCN-PDC1 (46) were digested, ligated together, transformed into *Escherichia coli*, and authenticated by Sanger sequencing (Genewiz).

### Broth microdilution assays

Four replicate colonies of each strain were grown to saturation overnight in SCD or SCD+ NAT (for plasmid-bearing strains) medium at 30°C. Resuspended cells were back-diluted 1:2,000 in fresh SCD or SCD+ NAT medium with 1.414-fold (root-2) serial dilutions of each antifungal in flat-bottom 96-well plates. Treated cells were grown for 20–24 h at 30°C, shaken, and OD_600_ was measured using an Accuris Instruments SmartReader 96T. The raw data were fitted to a sigmoid equation (Kaleidagraph), and the dose at which half-maximal growth is observed (IC50) was calculated independently for each replicate. IC50s were then used directly in *t*-tests or averaged and converted to log-2-fold change in comparison with controls. iKIX1 and ketoconazole were obtained from Cayman Chemicals.

### Relative gene expression with qRT-PCR

Replicate cultures of each strain were grown in liquid SCD medium at 30°C to early log phase and diluted to an OD_600nm_ of 0.1. For drug-treated samples, cultures were divided, and either oligomycin (20 µg/mL) or a vehicle control was added to the medium. Cells were pelleted after 1.5 h of shaking and flash-frozen in liquid nitrogen. The hot acid phenol method was used to extract RNA as done previously (38). The RNA was treated with DNase I and used to synthesize cDNA (Applied Biosystems cDNA Synthesis Kit, Cat. #4368814). Primers specific for *FKS1*, *FKS2*, *FKS3*, *CDR1*, *RTA1*, or *LAF1* (Table S3) were used to perform quantitative real-time PCR using ABsolute Blue qPCR Mix, SYBR Green (Cat. #AB4166), and a C1000 Touch thermal cycler (BioRad). *PGK1* and *TEF1* were used as internal controls in each sample.

### Statistical analysis

For drug resistance assays, four replicate IC50 values were compared to the parent strain or vector control using an unpaired two-tailed *t*-test. For qRT-PCR data, four replicate samples were compared using an unpaired two-tailed *t*-test for different strains, and a paired two-tailed *t*-test for drug-treated samples at time zero compared to 1.5 h of treatment.

## Data Availability

The authors affirm that all data necessary for confirming the conclusions of the article are present within the article, figures, tables, and repository. Raw sequencing reads used in this study were deposited at the NCBI Sequence Read Archive (SRA) with the BioProject ID PRJNA1247003. Micafungin treatment data for CBS138u and 2001u strains (27) were obtained from SRA BioProject ID PRJNA1133102. Fluconazole treatment data for strain BG2u (28) were obtained from SRA BioProject ID PRJNA625944. A tabulation of the chromosomal coordinates and frequency of each mapped transposon insertion site (site count files) and a tabulation of the number of mapped transposon sites that fall within an annotated gene boundary (gene count files) are available upon request.

## SUPPLEMENTAL MATERIAL

Figure S1 msphere.00270-25-s0001.tiff Z-score charts comparing all antifungals.

Figure S2 msphere.00270-25-s0002.tiff Growth measurements.

Table S1 msphere.00270-25-s0003.xlsx Z-scores from all experiments.

Table S2 msphere.00270-25-s0004.xlsx PCC from all experiments.

Table S3 msphere.00270-25-s0005.xlsx List of strains and oligonucleotides.

ASM does not own the copyrights to Supplemental Material that may be linked to, or accessed through, an article. The authors have granted ASM a non-exclusive, world-wide license to publish the Supplemental Material files. Please contact the corresponding author directly for reuse.
