# Supplementary figures and images for: Tn-seq screens in Candida glabrata treated with echinocandins and ibrexafungerp reveal pathways of antifungal resistance and cross-resistance
Source: mSphere. 2025 Jul 7;10(7):e00270-25. doi: 10.1128/msphere.00270-25 (PMC12306164; doi:10.1128/msphere.00270-25)

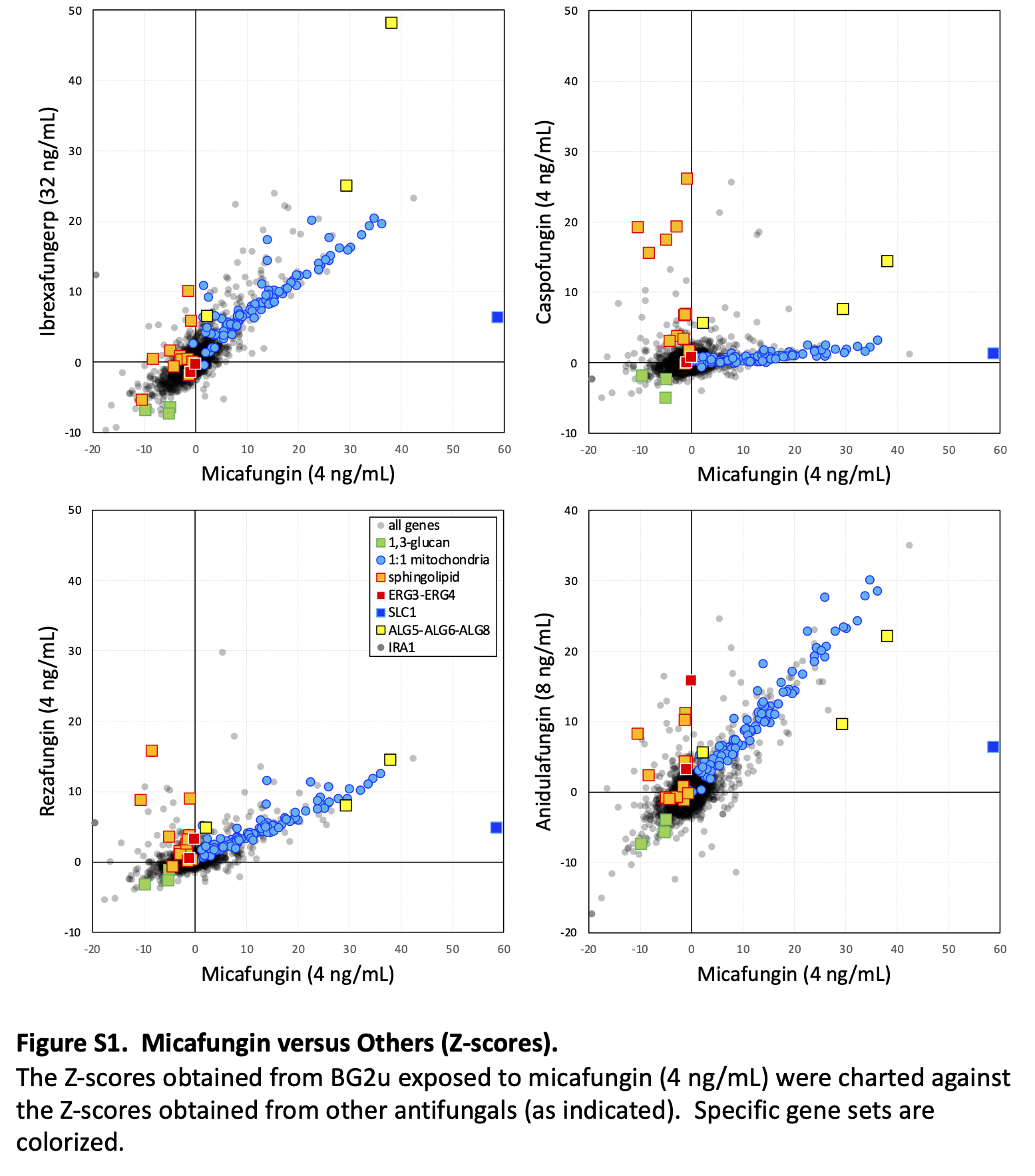

Supplement: Figure S1 — Z-score charts comparing all antifungals. [file msphere.00270-25-s0001.tiff]

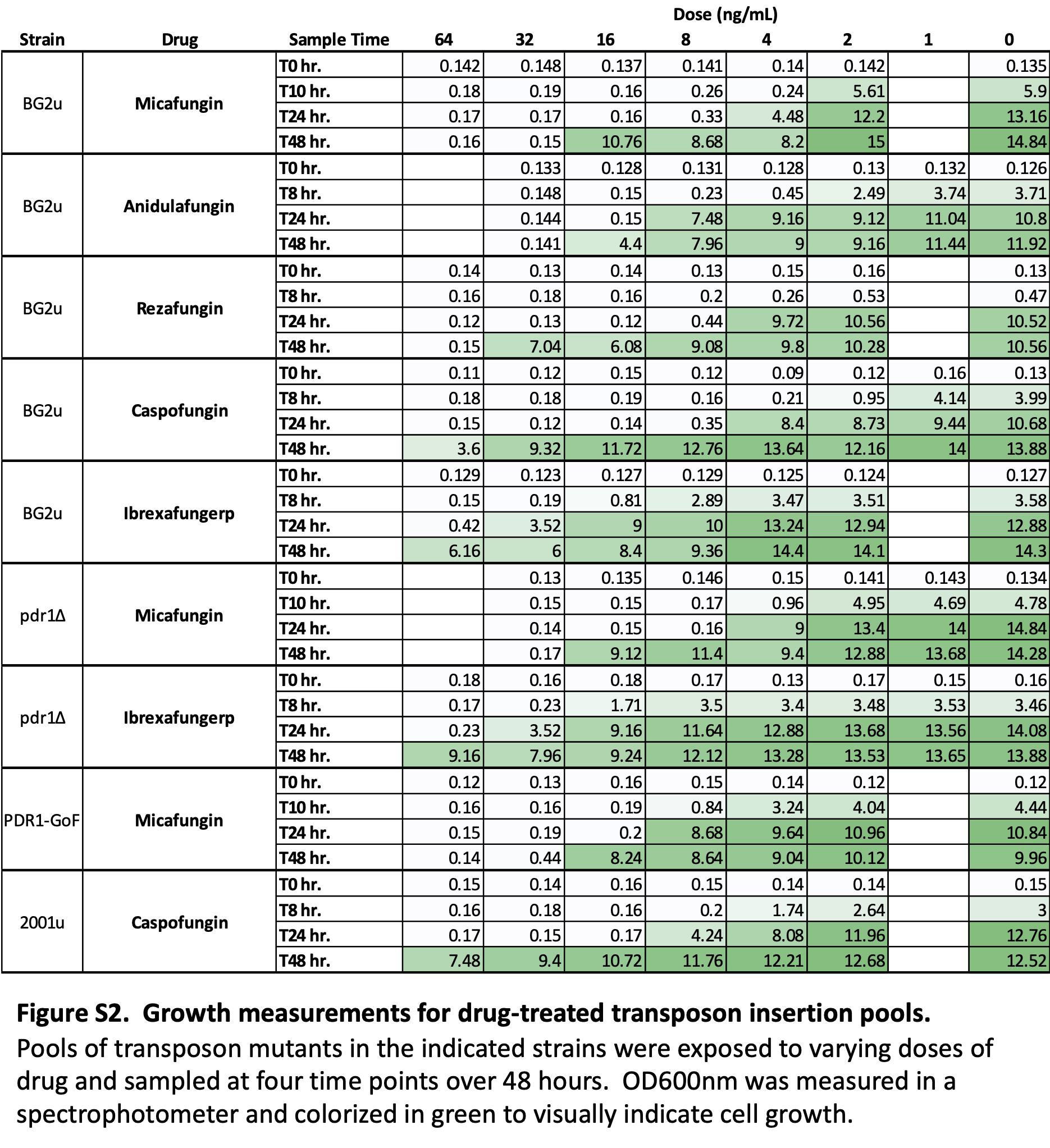

Supplement: Figure S2 — Growth measurements. [file msphere.00270-25-s0002.tiff]
